# Chromosome segregation of human nonhomologous Robertsonian translocations: insights from preimplantation genetic testing

**DOI:** 10.1038/s41431-024-01693-w

**Published:** 2024-09-28

**Authors:** Peter Benn, Katrina Merrion

**Affiliations:** 1 University of Connecticut Health Center, Farmington, CT 06030 USA; 2 Natera, Inc., San Carlos, CA USA

**Keywords:** Cytogenetics, DNA sequencing, Aneuploidy

## Abstract

Robertsonian translocations (robs) are associated with a high risk for unbalanced segregations. Preimplantation Genetic Testing (PGT) offers an early opportunity to evaluate segregation patterns and selection against chromosome imbalances. The objective of this study was to evaluate the chromosome complements in blastocysts for male and female rob carriers and provide information useful in PGT counseling for rob carriers. PGT results were reviewed for 296 couples where a balanced and nonhomologous rob was present in one member of the couple. All embryos had day 5/6 trophectoderm biopsy and SNP-based PGT. The study included 2235 blastocysts, of which 2151 (96.2%) had results. Significantly fewer blastocysts were available for female rob carriers (mean 4.60/IVF cycle) compared to males (5.49/cycle). Male carriers were more likely to have blastocysts with a normal/balanced chromosome complement; 84.8% versus 62.8% (*P* < 0.00001). Male carriers had fewer blastocysts with monosomy (60/152, 39.5%) compared to female carriers (218/396, 55.1%) (*P* = 0.001). Twenty-one (1%) blastocysts showed 3:0 segregation; these were mostly double trisomies and derived from female carriers. Differences between chromosome complements for male versus female carriers suggest that selection against unbalanced forms may occur during spermatogenesis. Six blastocyst samples showed an unexpected (“noncanonical”) combination of trisomy and monosomy. One case of uniparental disomy was identified. For female carriers, there was no association between unbalanced segregation and parental age but for male carriers, there was an inverse association. PGT is a highly beneficial option for rob carriers and patients can be counseled using our estimates for the chance of at least one normal/balanced embryo.

## Introduction

The short arms of the human acrocentric chromosomes (numbers 13, 14, 15, 21 and 22) contain ribosomal DNA repeats and other pseudo-homologous sequences. Their close proximity at interphase nucleoli and the presence of inverted sequences renders them susceptible to exchanges that are referred to as Robertsonian translocations (robs) [1]. These chromosome translocations are the most common specific rearrangements encountered in humans [2], with an overall frequency of approximately 1 in 800 individuals [3]. rob(13;14)(q10;q10) and rob(14;21)(q10;q10) are the most common types seen.

Although robs involve the loss of acrocentric short DNA sequencies, rob carriers with 45 chromosomes show no phenotypic consequences, and their karyotypes are often described as “balanced” and “euploid.” However, there are reproductive consequences. For those with robs involving homologs (homologous translocation), essentially all conceptions are expected to be trisomic or monosomic for the long arm sequences of the relevant chromosomes which is expected to result in either a nonviable embryo, lack of implantation, pregnancy loss, or specific syndromes at birth (notably, trisomy 21 (Down syndrome) and trisomy 13 (Patau syndrome)) [4, 5]. Carriers of robs involving nonhomologous chromosomes (heterologous translocation) can have normal offspring but are at increased risk for infertility [6], pregnancy loss [7], and, for those involving chromosomes 21 or 13, livebirths with Down syndrome or Patau syndrome, respectively [8].

Segregation of robs is based on the notion that translocated chromosomes are not able to pair correctly at meiosis 1 and a trivalent structure arises [9]. Chromosomes then segregate in an alternate pattern (resulting in normal or balanced gametes) or an adjacent pattern, resulting in either trisomy or monosomy (for either of the involved chromosomes) in the zygote. It is also possible that all 3 chromosomes segregate together which will result in double trisomy or double monosomy in the zygote.

Preimplantation genetic testing for aneuploidy (PGT-A) or for structural rearrangements (PGT-SR) is offered to couples where one parent is known to be a carrier of a rob, as this can potentially improve clinical pregnancy and outcomes [10]. Early studies on blastomere samples analyzed by fluorescence in situ hybridization (FISH) showed high levels of unbalanced translocations compared to blastocyst samples analyzed by microarrays, and this was interpreted as evidence for early selection against embryos with imbalances [11, 12]. More recent studies, mostly on blastocysts, have established that the proportion of normal/balanced translocation embryos is higher for male carriers compared to female carriers [13–18]. Previous studies have also shown that there is no parental age effect in the segregation of robs [13, 14, 19]. Jia et al. [15] concluded that there was no significant difference in the segregation rates for the different robs; however, this conclusion was based on a relatively small dataset.

In this study, we reviewed the chromosome complements of blastocyst samples from a large set of patients referred to a single commercial reference laboratory for PGT where the indication for testing was a balanced rob in one member of the couple. We provide evidence that segregation and/or selection results in chromosome complements in embryos that can be more complex than previously recognized. We also consider the clinical implications of our observations.

## Methods

This retrospective cohort study included PGT data from blastocyst samples received between April 2011 and May 2023 at a single reference laboratory with the indication of a parental balanced rob. Parental translocation type, as confirmed by cytogenetic karyotype analysis, was reviewed for each referred case. In vitro fertilization (IVF) cycles and day 5/6 trophectoderm biopsies on blastocysts were performed by the referring IVF clinics. Blastocyst biopsy samples and a biological sample from each member of couple pair were shipped for analysis. These latter samples were either blood, buccal swab, or sperm (typically in cases using a sperm donor). PGT for the parental translocation with concurrent 24 chromosome aneuploidy screening was performed using Illumina CytoSNP-12b microarrays with Parental Support bioinformatics, as previously described [20]. In brief, this method utilizes parental SNP genotype information to predict the possible SNP genotypes for an embryo. The testing algorithms compare the observed SNP data with the predicted allele distributions for different copy number hypotheses and identifies the maximum likelihood copy number for each chromosome. This analysis allows for detection of monosomy, trisomy or higher polysomy, haploidy, triploidy, large segmental deletions/duplications, uniparental disomy (UPD), and parental origin of abnormalities. This methodology does not allow for differentiation between an embryo that is normal (euploid) versus balanced. The analysis of the array information was not validated to distinguish between mosaic and nonmosaic chromosome abnormalities and results were therefore only classified as either normal/balanced or abnormal.

Data was compiled for each patient’s IVF/PGT cycle and included information on which rob was present, which parent was a carrier, parental ages at the time of testing, number of blastocyst samples analyzed, and the genetic findings. Results were classified as “unbalanced” if monosomy or trisomy of the correct parental origin was present for a chromosome involved in the rob and “normal” if no chromosome imbalance was detected. “Sporadic aneuploidy” was defined as aneuploidy involving chromosomes not involved in the translocation or involving a translocation homolog but derived from the parent not carrying the rob.

Statistical analyses were performed using online calculators. Differences in proportions were tested with the T-test for independent ratios (https://www.socscistatistics.com/tests/ztest/), Chi-square test was used for differences between grouped data (https://www.socscistatistics.com/tests/chisquare2/default2.aspx) with Fisher exact test for small numbers (https://www.socscistatistics.com/tests/fisher/default2.aspx), Mann-Whitney test for ordinal data (https://www.socscistatistics.com/tests/mannwhitney/), and Cochran-Armitage Chi-square test for linear trend (https://epitools.ausvet.com.au/trend). To evaluate the probability of at least one normal/balanced blastocyst in a specific number evaluated, a binomial distribution calculator was used (https://stattrek.com/online-calculator/binomial?utm_content=cmp-true). Confidence intervals were based on the adjusted Wald method (https://measuringu.com/calculators/wald/).

## Results

There were 296 couples referred for PGT because either the female (55.1%; 163/296) or the male (44.9%; 133/296) partner was a carrier of a nonhomologous rob. Table 1 summarizes the tested translocations, carrier parent ages at the time of testing, utilization of repeat cycles, re-biopsy samples tested, and the rate of testing without results (due to insufficient DNA or uninformative SNP data).

**Table 1:** Types of robs, number of cycles, patient age, blastocysts for the study population.

	All rob carriers	Female carriers	Male carriers
Total patients	296	163	133
rob(13;14)	198	106	92
rob(13;15)	11	7	4
rob(13;21)	6	0	6
rob(13;22)	6	1	5
rob(14;15)	8	6	2
rob(14;21)	48	32	16
rob(14;22)	9	6	3
rob(15;21)	4	1	3
rob(15;22)	5	3	2
rob(21;22)	1	1	0
Total cycles	448	254	194
Without a repeat cycle	299	167	132
Repeat cycles	149	87	62
Number re-biopsies^a^	16	10	6
Mean maternal age^b^	34.8	34.8	34.8
Mean paternal age^b^	37	36.9	37.2
Blastocysts with results	2151	1126	1025
Blastocysts without results	84	43	41

^a^Re-biopsy was performed for a subset of embryos that did not produce a result on the initial biopsy sample. Results were based on the second (successful) analysis.

^b^Age at the time of the in vitro fertilization cycle. Age was re-calculated when there was a repeat cycle.

Table 2 summarizes the average number of blastocysts analyzed per cycle for rob(13;14), rob(14;21), and all robs combined. There were significantly fewer blastocysts when the carrier was maternal compared to paternal (average 4.60 versus 5.49 per IVF cycle, respectively), with the deficit largely associated with rob(13;14). This did not appear to be attributable to age-related decline in female fertility; there was no significant difference between the age of female carriers and females with a carrier partner (mean 34.84 years versus mean 34.81 years, *P* = 0.873).

**Table 2:** Blastocysts per cycle for female and male carriers of Robertsonian translocations.

	Cycles	Blastocysts^a^	Average Blastocysts/cycle	Female vs Male carriers *P*^*b*^
All rob				
Female carriers	254	1169	4.60	*P* = 0.038
Male carriers	194	1066	5.49	
rob(13;14)				
Female carriers	168	773	4.60	*P* = 0.016
Male carriers	133	746	5.61	
rob(14;21)				
Female carriers	44	237	5.39	*P* = 0.865
Male carriers	23	131	5.70	

^a^Includes blastocysts without successful analysis.

^b^Mann Whitney Test for the number of blastocysts in each cycle.

The segregation products in the blastocysts with successful analysis are summarized in Table 3. Male carriers were significantly more likely to have blastocysts with a chromosomally balanced chromosome complement (alternate segregation); balanced rates were 84.8% for male carriers versus 62.8% female carriers (*P* < 0.00001). Subset analysis restricted to rob(13;14) and rob(14;21) showed that the higher frequency of balanced products for male carriers was present for each of these more common rob types.

**Table 3:** Summary of the observed segregation products in the blastocysts from rob carriers.

	Segregation	Female carriers	Male carriers
		Blastocysts (%)	
All rob			
	All	1126 (100)	1025 (100)
	Alternate	707 (62.8)	869 (84.8)
	All adjacent	396 (35.2)	152 (14.9)
	Monosomy	218 (19.4)	60 (5.9)
	Trisomy	178 (15.8)	92 (9.0)
	3:0	17 (1.5)	4 (0.4)
	Noncanonical	6 (0.5)	0 (0)
rob(13;14)			
	All	743 (100)	720 (100)
	Alternate	475 (63.9)	617 (85.7)
	All adjacent	251 (33.8)	103 (14.3)
	Monosomy 13	61 (8.2)	23 (3.2)
	Trisomy 13	48 (6.5)	34 (4.7)
	Monosomy 14	79 (10.6)	13 (1.8)
	Trisomy 14	63 (11.5)	33 (4.6)
	3:0	12 (1.6)	0 (0)
	Noncanonical	5 (0.7)	0 (0)
rob(14:21)			
	All	228 (100)	125 (100)
	Alternate	133 (58.3)	101 (80.8)
	All adjacent	93 (41.0)	24 (19.2)
	Monosomy 14	9 (3.9)	6 (4.8)
	Trisomy 14	10 (4.4)	4 (3.2)
	Monosomy 21	41 (18.0)	6 (4.8)
	Trisomy 21	33 (14.5)	8 (6.4)
	3:0	1 (0.4)	0 (0)
	Noncanonical	1 (0.4)	0 (0)

Table 3 also shows the breakdown of monosomy versus trisomy in the segregations interpreted as adjacent. For all robs combined, the proportion of blastocysts that had monosomy was significantly lower for male carriers (60/152, 39.5%) than for female carriers (218/396, 55.1%) (test for two independent proportions, *P* = 0.001). However, this difference was dependent on the specific type of rob. For rob(13;14), the same pattern was observed; less monosomy was seen for chromosomes 13 or 14 for male carriers (36/103, 35.0%) compared to monosomy of chromosomes 13 or 14 for female carriers (140/251, 55.8%) (*P* = 0.0004). For rob(14;21), no such difference was evident with monosomy of chromosome 14 or 21 for male carriers (12/24, 50%) versus monosomy of chromosome 14 or 21 for female carriers (50/93, 53.8%) (*P* = 0.74).

There was also evidence for nonrandom segregation products for the 21 blastocysts with chromosome complements consistent with 3:0 segregation (16 with double trisomy and 5 with double monosomy). For maternal carriers, 16 cases of apparent 3:0 segregation involved double trisomy and one case showed double monosomy. In contrast, no double trisomy was observed for paternal carriers, but four double monosomies were detected (Fisher exact test, *P* < 0.05).

In six blastocysts (0.7%), there was a combination of a trisomy and a monosomy that was incompatible with the eight theoretically possible segregation products that can arise during segregation of a rob. We refer to these as “noncanonical”. All six were products from maternal carriers, with five involving rob(13;14) with trisomy 13 and monosomy 14, and one involving rob(14;21) with trisomy 14 and monosomy 21. There was only one case of UPD (heterodisomy) observed in our dataset. This case involved a male carrier of a rob(15;21) with an arr [15]x2 htz mat blastocyst sample.

To provide robust data for genetic counseling for individuals with rob(13;14) and rob(14;21), we combined our observations on the chance of a balanced segregation with the cases from two other recent papers (Table 4). We then calculated the probability of at least one normal blastocyst using a binomial calculator, under the assumption that each embryo was fully independent of others from the same patient. These observations showed that for the average referral with four to six blastocysts available (Table 2), PGT should substantially increase the chance of being able to select for a balanced embryo. These calculations did not include sporadic chromosome abnormalities unrelated to the rob.

**Table 4:** Overall chance for a normal/balanced (alternate) segregation for rob carriers with calculation of at least one normal/balanced segregation when multiple blastocysts are available.

Reference	Segregation	Females with rob(13;14)	Males with rob(13;14)	Females with rob(14;21)	Males with rob(14;21)
Zhang et al. [14]	All	261	314	45	87
	Alternate	183	260	37	50
	Adjacent	77	53	7	35
	3:0/others	1	1	1	2
Dang et al. [19]	All	968	1059	395	191
	Alternate	596	869	219	165
	Adjacent	365	186	167	26
	3:0/others	7	4	9	0
Current study	All	743	720	228	125
	Alternate	475	617	133	101
	Adjacent	251	103	93	24
	3:0/others	17	0	2	0
All studies combined	All	1972	2093	668	403
	Alternate	1254	1746	389	316
	Adjacent	693	342	267	85
	3:0/others	25	5	12	2
Overall proportion Alternate %, (95% CI)		63.6	83.4	58.2	78.4
		(61.3-65.5)	(81.8-85.0)	(54.4-61.8)	(74.1-82.2)
Probability (%) of ≥ 1 balanced blastocyst^a^:					
1 blastocyst available		63.6	83.4	58.2	78.4
2 blastocysts available		86.6	97.2	82.4	95.3
3 blastocysts available		95.1	99.5	92.6	99
4 blastocysts available		98.2	>99.9	96.9	99.8
5 blastocysts available		99.3	>99.9	98.7	>99.9

Does not include risk for a sporadic abnormality unrelated to the translocation.

**Fig. 1:**
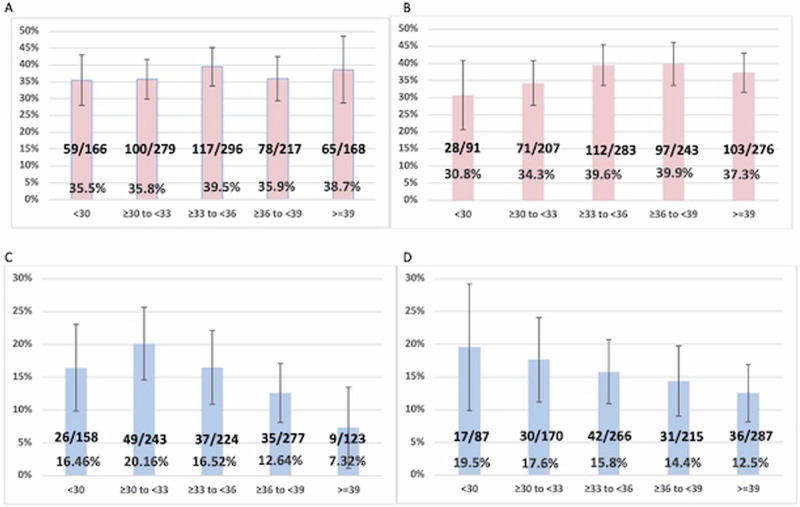
Proportion (%) of cases with unbalanced segregation products and parental age. x-axis, age group; y-axis proportion (%) with unbalanced segregation. **A** Maternal age for female carriers. **B** Paternal age for maternal carriers. **C** Maternal age for male carriers. **D** Paternal age for male carriers. Vertical lines denote 95% confidence intervals.

To explore a potential association between carrier age and segregation patterns, the data for female and male carriers was separately analyzed, with data grouped into 3-year age ranges. Figure 1A shows that there is no trend in the rate of unbalanced segregations for maternal carriers across the different maternal age groups (Armitage-Cochran test, *P* = 0.58, slope = 0.006). Since maternal and paternal ages are correlated, the observed lack of an age association seen for paternal age was expected and seen (*P* = 0.23, slope = 0.014) (Fig. 1B). To further look for evidence of a maternal age effect, we considered only 3:0 segregations from female carriers. For the 17 cases (16 cycles) with 3:0 segregation in blastocysts from female carriers, the mean maternal age at the time of testing was 34.6 years compared to 34.8 years for female carriers with other types of segregation (Mann-Whitney test, *P* = 0.624).

For paternal carriers, there was a trend toward lower rates of unbalanced segregations with increasing parental age (Fig. 1C, D). For paternal age, this did not reach statistical significance (*P* = 0.052, slope −0.017) but for maternal age, a stronger trend was observed (*P* = 0.0035, slope −0.026) which differed significantly from linearity and indicated a likely nonlinear relationship.

To assess whether unbalanced robs were associated with increased levels of other chromosome abnormalities (interchromosomal effect) [10, 21], or reduced levels due to reduced viability of embryos with multiple chromosome imbalances, we assessed the rate of sporadic chromosome abnormalities (i.e., unrelated to the rob). When the embryo had an unbalanced rob, the rate of sporadic abnormalities was 38.7%. For those embryos that were balanced or normal for the rob chromosomes, the rate of sporadic abnormalities was 42.8%. The difference between the two rates did not reach statistical significance (*P* = 0.086). For a maternal age-matched control population (excluding egg-donors) that were not translocation carriers, the rate of sporadic abnormalities was 44.1%. These data do not fully exclude an interchromosmal effect involving specific chromosomes (for example, acrocentrics not involved in the translocation).

## Discussion

In this study, we reviewed the chromosome complements of embryos from individuals referred for PGT-SR because either the male or female was known to be a carrier of a rob. We show that the chromosomes observed did not necessarily correspond to the expected classical view of how segregation of robs occurs. Specifically, we document trisomy plus monosomy, higher than expected monosomies compared to trisomies, and differences in 3:0 segregation (double trisomy versus double monosomy) depending on the sex of the carrier parent. We also provide relatively robust estimates for the probability of at least one chromosomally balanced embryo when PGT is offered.

Contemporary views of chromosome segregation at meiosis recognize that precocious separation of chromatids, anaphase lag, reverse segregation, and nondisjunction have a role in the formation of aneuploidy [22]. In addition, high rates of early mitotic error (zygote or earliest embryonic divisions) are present [23]. The propensity for individual whole chromosomes to segregate abnormally at meiosis appears to be inversely correlated with chromosome size [24]. Longer chromosomes have more cohesin holding chromatids together and this is thought to minimize the chance for segregation error [22]. Precocious separation of chromatids is strongly maternal-age dependent while the nondisjunction mechanism, at least for the acrocentric chromosomes, is less dependent on maternal age [25]. Our observations of no maternal age effect in the segregation for unbalanced robs can perhaps be explained by unbalanced translocation segregation being a similar process to nondisjunction. Alternatively, the heterozygous change in length associated with a rob and the presence of the associated additional cohesin might be sufficient to substantially reduce maternal-age related segregation error. Precocious separation of chromatids, anaphase lag, reverse segregation, and mosaicism may explain the noncanonical chromosome segregation patterns we observed in rare cases.

Our observation of differences in the number of embryos, and differences in the chromosome complements, depending on which parent is a carrier, might be explained by basic differences in the meiotic processes in males versus females. Supporting this theory, it has previously been pointed out that even for chromosomally balanced segregations, there appears to be differences in the proportions of normal versus balanced translocation karyotypes [26–28]. The excess of rob carriers over normal karyotypes cannot be easily explained by post-meiotic correction of unbalanced segregations. Early somatic cell gain or loss of a chromosome to correct from monosomy or trisomy to disomy is rare; we observed only one case with UPD which can arise due to correction [29]. Post-meiotic selection against unbalanced chromosome complements in spermatogenesis is also possible and this might explain the lower proportion of unbalanced embryos for male carriers compared to female carriers. Male carriers of robs have increased rates of infertility and may show reduced sperm counts, perhaps reflecting the selection against imbalances, notably spermatids and sperm with nullisomy [30]. Our observation of higher rates of alternate segregation (and an excess of trisomy relative to monosomy), for the blastocysts from male carriers would be consistent with this explanation. FISH studies on sperm from rob carriers have shown more alternate segregation gametes compared to adjacent segregation gametes [31]. We also observed lower rates of unbalanced robs in the blastocysts for older male carriers, suggesting that the selection processes in males may also be affected by other factors that reduce sperm counts [32].

Selection against embryos and fetuses through miscarriage further reduces the relative proportion of unbalanced conceptions. Most autosomal monosomic conceptions are believed to be lost very early in pregnancy, and surviving autosomal trisomies depend on the gene imbalance with highest survival for trisomies 21, 18, and 13 [33]. It is therefore useful to compare ratios of trisomy to normal/balanced in blastocysts versus later in pregnancy. For example, for rob(13;14) the ratio of trisomy 13 to normal/balanced blastocysts for female carriers was 48:475 (approximately 1:10) and for male carriers was 34:617 (approximately 1:18) (Table 3). In the second trimester, trisomy 13 arising from a rob(13;14) is approximately 1–2% [25]. This level of reduction from embryo to the second trimester is surprising given that trisomy 13 can potentially survive to birth. In the case of rob(14;21), the ratio of trisomy 21 to normal/balanced karyotypes in blastocysts from female carriers was 33:133 (approximately 1:4) and for male carriers 8:101 (approximately 1:8) (Table 3). In the second trimester, risk for trisomy 21 has been estimated to be 12:80 (approximately 1:7) for females but only 1:37 for male carriers [25]. More robust data is needed to better establish whether or not there is a differential effect on the viability of an embryo or pregnancy depending on the parent of origin of the chromosomes.

Our observations have direct clinical application. We show that PGT is highly advantageous to individuals with robs because, in most instances, there will be sufficient normal/balanced embryos (Tables 2 and 4). Strong differences exist between female versus male carriers, and risk for a surviving fetus with an imbalance will differ depending on the specific rob. Although male carriers of robs often show reduced sperm count and lower sperm mobility, they can be assured that the potential for an embryo with a normal/balanced segregation is relatively high. Probabilities for a normal blastocyst can be separately provided for rob(13;14) and rob(14;21). We suggest that for the rarer rob types, counseling is based on all rob combined, at least until more information is available on their segregation patterns. Caution is needed because these other forms may, at least in part, be rarer because of a higher rate of unbalanced gametes. In an era where most chromosome abnormalities are identified through molecular techniques that do not identify balanced translocations, our study serves as a reminder of the clinical value of ruling out rob segregation when Down syndrome and Patau syndrome is diagnosed.

A strength of this study was the use of SNP microarray technology which included parental genotype information to determine the origin of each chromosome in the blastocysts. Thus, our data provided the ability to differentiate whether a chromosome abnormality involving one of the chromosomes of the rob was from the carrier parent (unbalanced segregation) vs. noncarrier parent (an unrelated sporadic aneuploidy), and to identify UPD. Our study was based on a large set of referrals which allowed us to separately analyze rob(13;14) and rob(14;21).

Our study also has some limitations. The molecular genetic approach was not used to distinguish between the two possible alternate segregation patterns, normal versus a balanced translocation. How the carriers were initially ascertained (e.g., infertility, history of spontaneous abortion(s), an affected child, or secondary finding in cytogenetic studies for other indications) is unknown and it is possible that the cases referred are not representative of all rob carriers in the population. Information on the use of ovarian stimulation, notably for female carriers [34], number of oocytes retrieved, and other clinical or laboratory data that might have affected referrals or IVF success rates was not available to us. Our interpretation of embryos showing segregation patterns is based primarily on the classical expectations and it is possible that, in addition to the above noted noncanonical examples, more imbalances could be attributable to precocious chromatid separation, anaphase lag, reverse segregation, or an early mitotic error. This study does not include a control population to assess the number of embryos for noncarrier couples. Additionally, when comparing rates in males versus females, we did not consider the (relatively minor) difference in age in carrier females versus carrier males.

In summary, we have provided evidence that the segregation for robs is more complex than previously recognized. In addition to asymmetric segregation at meiosis, there may be complex selection processes from the time of the initial meiotic segregation to the time of early embryo development. We demonstrate the benefit of PGT as a reproductive testing option for rob carriers.

## Data Availability

Data will be made available to upon request from Z Demko, Natera, Inc. zdemko@natera.com.
